# Leveraging university-industry partnerships to optimise postgraduate nursing education

**DOI:** 10.1186/s12912-023-01419-1

**Published:** 2023-08-04

**Authors:** Karen A. Theobald, Robyn Fox, Christine Burridge, Bernadette Thomson, Amanda Fox

**Affiliations:** 1 School of Nursing, Queensland University of Technology, Victoria Park Road, Kelvin Grove, Brisbane, Australia; 2 Metro North Hospital and Health Service, Brisbane, Australia; 3 Metro South Health, Brisbane, Australia; 4 Queensland University of Technology, Brisbane, Australia

**Keywords:** Postgraduate education, Professional education, Co-design, Partnerships, Sustainability

## Abstract

**Background:**

Industry and higher education sectors devote considerable, but independent resources to deliver postgraduate nursing education. This leads to duplication, uncertainty among students, and critical gaps in nursing education. Establishing and sustaining meaningful partnerships between invested university and industry stakeholders can strengthen workforce capability and improve patient care.

**Methods:**

To evaluate the feasibility and effectiveness of using a University-Industry Integration Framework to develop a postgraduate nursing education program. Prospective mixed methods cohort study (STROBE). A co-design approach, using an established University-Industry Integration Framework, leveraged expert stakeholder partnerships to contextualise knowledge and service need for developing a postgraduate education program for cancer care nurses.

**Results:**

All participants (n = 46) were 100% satisfied with the online resources, support, and communication processes applied. Qualitative data generated three major analytical interpretations (reciprocity, flexible adaptations, authentic learning), highlighting the experiences and connections and how the partnership evolved. Program participants (n = 15) undertook a six-week cancer education program with eight responding to the survey with overwhelming satisfaction (100%), increasing their knowledge and skills. While barriers were evident, three quarters (n = 6) indicated these were addressed and enabled progress in the program. However, 63% (n = 5) were not satisfied with the program workload.

**Conclusions:**

University and industry partners can apply the University-Industry Integration Framework and deliver a successful postgraduate education program for cancer care services. Within a co-design partnership it is possible to develop strategies and processes to overcome barriers and deliver a program for mutual benefit. The culmination of this successful education program has enhanced collaborations between partners and likely will sustain the offering of future co-design endeavours.

**Supplementary Information:**

The online version contains supplementary material available at 10.1186/s12912-023-01419-1.

## Background

Health systems depend on the specialised knowledge of nursing staff and high-functioning, team-based approaches to deliver person-centred care. Postgraduate professional education should facilitate an educated health workforce and research-literate nurses who can implement evidence-based practice within scope of regulatory frameworks and organisational requirements [1–3]. Evidence suggests that increased education for nurses leads to increased confidence, communication, analytical thinking and decision making [4]. Audet and colleagues [5] report that higher levels of nurse education and experience are associated with reduced mortality and lower levels of adverse events for patients. Billett’s early Australian Learning and Teaching Council Fellowship work acknowledged the contribution of work-integrated education, when designed and delivered effectively, significantly promotes an individual’s professional learning [6]. Learning is prefaced on the development of appropriate teaching that is delivered through academic, discipline and industry specific knowledge, skills and attitudes [3, 7]. Therefore, it is effective practice for universities and health industries to build formal partnerships to develop, deliver, and evaluate ongoing context specific postgraduate education.

Research suggests that post-registration nurses benefit from a judicious blend of practical and theoretical learning experiences [8]. Such experiences rely on robust and coherent approaches to postgraduate curricula that maximise enablers and minimise barriers to university and industry partnerships. Creating formal integration of clinical-academic enterprise in nursing should enable the delivery of a focused, compelling, collaborative, unified and mutually accountable program of postgraduate learning [3, 7].

University and industry sectors invest significant but separate financial and human resources to deliver postgraduate nursing educational programs [9]. This education ranges from purely practical to intensely theoretical, and from short continuing professional development modules to programs developed to meet award course requirements. This approach often results in repetition of curricula development, delivery and resource financing across both sectors [1]. Award courses are those which lead to a recognised qualification and are governed according to varied tertiary education agencies. In Australia, tertiary education providers are governed by the Tertiary Education Quality and Standards Agency (TEQSA) [10]. Further, the national Australian Qualifications Framework (AQF) regulates policy for all education and training provision [11]. Overseas in the United States, graduate programs are delivered by over 4,300 higher education and university providers [12], of which some are grouped under the Association of American Universities membership, like the United Kingdom’s Russell Group, and are badged to ensure quality and collective voice with issues in higher education [12]. University and the industry sector bring complementary contributions to nursing curriculum development; however, each is driven by different organisational imperatives, resulting in divergent goals, approaches and different metrics to evaluate educational value [1, 3].

The University-Industry Integration Framework [2] recommends strategies to build collaborative relationships among university, industry, education and professional bodies for postgraduate education to sustain ‘work ready’ specialist nurses. The project explored how collaborative relationships may be embedded within an integrated partnership framework to support co-designed curricula. The framework claims that progressing a shared culture of curriculum development supports university and industry experts to work in a co-design partnership to develop a mutually agreed professional learning experience [3]. This work provides a foundation for collaboration and capacity-building between university and industry sectors. This study has used the framework and recommendations to co-design and deliver a program within Cancer Care Services.

## Methods

### Aim

The aim of this study was to evaluate the feasibility and effectiveness of the University-Industry Integration Framework [2] when applied to co-develop and deliver a postgraduate nursing education program in a cancer care setting.

The research questions are:

1. Is it feasible for university and industry partners to use the University-Industry Integration Framework [2] to co-design and deliver a postgraduate educational program for cancer care nurses?
2. What strategies and processes enabled and/or hindered university and industry stakeholders to collaborate, develop, implement, participate and evaluate the educational program?
3. What were the Educational Program Partnership Group’s and program participants’ experiences of a co-designed postgraduate educational program?

### Design

This prospective cohort study employed mixed methods to evaluate a co-design approach and framework used to develop, evaluate and deliver a postgraduate nurse education program. This approach drew on Sanders and Stappers [13] conceptualisation of co-design, used previously by the lead author in developing the original framework evaluated in this study [3]. Guidelines for reporting observational studies (cohort) using the STROBE checklist have been applied.

### Setting

This research was conducted over six months across two large metropolitan health services in Queensland, Australia and in the School of Nursing of a large metropolitan university.

### Participants

The purposeful sample of program participants were registered nurses (RNs) who were beginning practitioners working in cancer care services at the participating sites. As this program aimed to support beginning cancer nurses to transition safely into the workplace, RNs who possessed a postgraduate qualification in cancer were excluded. Industry partners advised that new staff would need at least three months experience to meaningfully engage with the program.

The Educational Program Partnership Group comprised key personnel from each site. The *Project Team* were a group of stakeholders from each organisation responsible for conceptualising and developing the agreed educational program; An *Advisory Committee*, who provided high-level strategic direction and input on program deliverables; a *Working Group* of industry managers, nurse educators, university lecturers, academic leaders and researchers who provided expert advice to co-design, develop, implement and evaluate the program. *Directors of Nursing* from each industry site who authorised the nurses’ program participation.

### The education program

The postgraduate educational program was designed to advance knowledge and professional skills among participating nurses and provide opportunities for them to demonstrate autonomy and judgement as an introduction to the specialty of cancer nursing. The program was delivered over six weeks via modules located in a Blackboard Learning Management System. The modules taught Population Health, Cancer Biology, Classisfication, and Diagnosis. Learning was self-directed and self-paced, requiring program participants to invest approximately eight to 10 h per week. Modules were comprised of: learning objectives; core theoretical content, with reference to literature; and a range of interactive blended learning activities. Three synchronous webinars were delivered to support program participants’ online learning and were facilitated by industry nurse educators and university lecturers. Program participants were encouraged to complete the two assessment activities, a case study, and a brief oral presentation of the case.

### Recruitment and consent procedures

Identification of potential program participants occurred over a two-month period through line managers and nurse educators who were able to identify all new staff who met the inclusion criteria. The Senior Research Assistant conducted information sessions and provided the participant information sheet and consent forms. Written consent was also obtained from the Educational Program Partnership Group. Completed consent forms certified participation in the study and subsequent provision of evaluation specific to each Educational Program Partnership Group (i.e. semi-structured interviews, surveys, reflective notes and action meeting notes). The decision to participate was voluntary and participants could withdraw at any time without consequence. All data was confidential and deidentified at data collection. Recruitment, engagement, follow up and data collection occurred over a six-month period (1 January 2021–31 June 2021).

### Data collection

Data included transcribed audio recordings of semi-structured interviews, reflective notes, action meeting notes and surveys conducted between February and June 2021.

#### Interviews

Semi-structured interviews with the Project Team and Directors of Nursing explored the co-design and collaborative process of achieving mutual understanding in developing and delivering the education program. The Senior Research Assistant conducted interviews in a private room (face-to-face) or online. Recorded audio and data were anonymised prior to transcription. Interview questions included, How did you reach common goals? What are your views on sustainability? and What makes a successful education program?

#### Reflective notes

To support reflection in action and reflection on action [14], program participants were requested to write a reflection at two time points (middle and end of the program), outlining their experiences of participating in the educational program. These were de-identified and confidential.

#### Action meeting notes

To gain an appreciation of engagement and interaction, action notes were collated from the Project Team, Advisory Committee, and the Working Group stakeholders’ meetings. These notes assembled information related to tasks, actions, structures, and processes, which enabled or hindered collaboration and integration. These notes were de-identified and confidential.

#### Survey

Surveys were used for the program participants, Advisory Committee, Working Group and Director of Nursing stakeholders to gain an understanding of the level of satisfaction with involvement and perceived relevance of the final education program. Surveys were developed by the research team and consisted of eight core questions with a five-point Likert scale for responses ranging from very satisfied, satisfied, neutral, dissatisfied and very dissatisfied. Respondents were also able to add comments to an open-ended section.

### Data analysis

#### Qualitative analysis

Qualitative analysis followed a critical and iterative review of data generated from the semi-structured interviews, reflective notes and action meeting notes [15]. Each data source (audio recordings, interview transcriptions, reflective notes, and action meeting notes) was detailed, described, and collated. Interview analysis commenced with familiarisation of data through close listening to audio recordings, then line-by-line reading of transcriptions. Familiarisation with the data occurred via an iterative process that included noting unusual and interesting points, and observing repetition, exceptions, contradictions, and ambiguity [15]. This enabled: comparison of data; exploration of associations; assigning preliminary codes to data in order to describe the content; searching for patterns or themes across the different data sources; and defining and developing analytical points. This process of considering words, phrases, concepts and annotation of the materials assisted in the explaining, clarifying and making of inferences as the analytical interpretations evolved.

#### Quantitative analysis

Survey data was entered into IBM SPSS Statistics (version 27) [16]. Descriptive statistics, frequencies, and percentages were used to examine the characteristics of the sample. Data was entered, coded, cleaned and analysed following standard processes. Likert scale responses were condensed into three categories: dissatisfied (very dissatisfied and dissatisfied responses); neutral; and satisfied (very satisfied and satisfied responses).

#### Data integration

Data integration was guided by the research questions, with the quantitative findings synthesised according to the themes generated from the qualitative data. The quantitative and qualitative data combined subsequently informed the synthesised narrative of results in the discussion and conclusions. Trustworthiness was adhered to in this study based on Sandelowski’s [17] argument that this occurs through making research practices visible and therefore auditable. The practices of the research Project Team, all experienced quantitative and qualitative researchers, meeting regularly and sharing updates and outcomes supported this. Working regularly with the expert Advisory Committee in providing updates about research processes and outcomes supported accurate representation of findings and addressed issues early throughout the research.

## Results

A total of 15 program participants (RNs) consented to undertake the six-week cancer educational program. All 15 participants accessed the online educational platform at least once during the program. Two participants did not engage in the program after week three. Thirteen (86.7%) participants presented a case study online for peer and educator feedback during the program. One participant, citing family reasons, withdrew immediately prior to the final summative assessment. Three other participants chose not to submit the final assessment. At the program’s completion, nine participants submitted and passed the summative assessment.

Of the 15 participants who took part in the cancer educational program, eight (53%) responded to the survey. See Tables 1 and 2. Overwhelming satisfaction was reported (n = 8, 100%) for their involvement in the educational program: the program assisted to increase their knowledge and skills in cancer care, and content was relevant and aligned to the learning objectives. Likewise, participants were satisfied (n = 8, 100%) with the online resources, with support provided by the educators, with communication processes used, and that assessment tasks supported their learning during the program. Three quarters of the respondents (n = 6) were satisfied that barriers identified were addressed to allow them to progress in the program. Participants were not satisfied (n = 5, 62%) with their ability to manage the workload associated with the program.

**Table 1:**
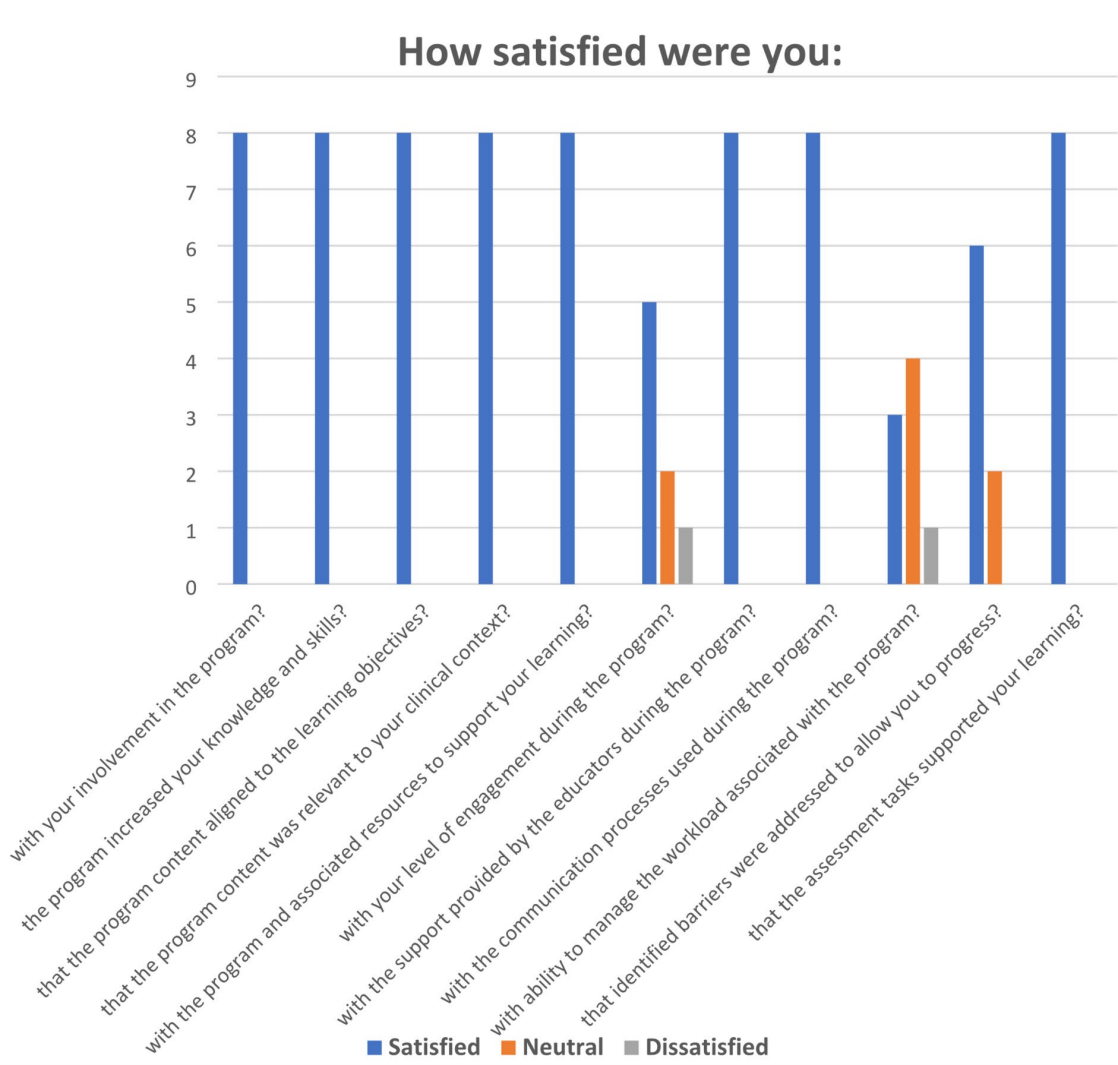
Program participant satisfaction

The Educational Program Partnership Group consisted of 31 members, of which 25 completed the survey, providing a response rate of 80%. Results indicated that the majority (n = 20, 80%) were satisfied that mutual understanding was realised within the group and that involvement in the program contributed to enhanced collaboration between university and industry partners. Respondents were strongly satisfied with their involvement in the project (n = 18, 72%) and contribution to building staff capability (n = 19, 76%). Satisfaction with the educational program and supportive resources was reported by 18 (72%) participants. Respondents were less certain about the ability of the educational program to be replicated in the future with 11 (44%) not satisfied that this would be possible. Likewise, 10 (40%) respondents were not satisfied that barriers that arose during the project were sufficiently addressed to progress the program. A total of seven (28%) respondents were not satisfied with the communication processes used during the project. Of these, four (57%) were from the Participant Industry Manager or were Nurse Educators; two (28%) were from the Working Group; and one (14%) was from the Advisory Committee. See Table 2 below.

**Table 2:** Educational Program Partnership Group satisfaction (n = 25)

How satisfied were you:	Satisfied n (%)	Neutral n (%)	Dissatisfied n (%)
with your involvement in the project?	18 (72)	4 (16)	3 (12)
that a process of mutual understanding was realised by the team and other key stakeholders?	20 (80)	2 (8)	3 (12)
with the online product and associated resources offered to support the project?	18 (72)	3 (12)	4 (16)
with the communication processes used during of the project?	17 (68)	1 (4)	7 (28)
that identified barriers were addressed to progress the intent of the collaborative processes of the project?	15 (60)	2 (8)	8 (32)
that the educational program can be replicated for future application?	14 (56)	7 (28)	4 (16)
that your involvement in this project has contributed to building capacity?	19 (76)	4 (16)	2 (8)
that your involvement in this project has contributed to enhanced collaboration?	20 (80)	2 (8)	3 (12)

A sub-set of questions were asked of the Participant Industry Managers, Nurse Educators and.

Lecturers (n = 10) with a 70% (n = 7) response rate. The majority (n = 4, 57%) were not satisfied with their engagement with participants, the level of engagement by program participants (n = 4, 57%), or their ability to manage the workload associated with the project (n = 4, 57%).

### Qualitative data

The findings from all qualitative data in this study were synthesised, (seven semi-structured interviews, up to two reflective notes per trial participant and 55 action meeting notes) and generated three major analytical interpretations, with a focus on the partnership process that evolved between university and industry during the study. The interpretations overlap demonstrating interconnectedness and complexity, as the partnering relationships changed.

#### Reciprocity

The process of establishing mutual understanding commenced through the natural alignments and familiarity of three stakeholders with existing relationships. Building on these relationships, the new Project Team expanded to five members who explored a shared concern for contemporary graduate capabilities and what nursing practice entails. Identifying this enabled the group to consider the different perspectives of how education could be developed to suit the desired outcomes of university and industry stakeholders. They recognised the current context of duplication was unproductive, unsustainable and did not meet student needs.

We can’t keep doing what we are doing … it is counterproductive. (Project Team Interview 3)

Setting a platform of collegiality was crucial for these beginning conversations, as the Project Team focused on new ways of working together established on trust, authenticity, and openness to ideas. The ability to speak up, as well as the acceptance of respectful, robust discussion and appreciation of previous knowledge and expertise developed through the co-design process. An intentional flattened hierarchy enabled the Project Team to collaborate as “equal partners with equal responsibility” (Project Team Interview 3). Members became active and accountable by working with their strengths. Despite each stakeholder operating on different sites, “nothing was done in isolation” (Project Team Interview 5). Their sharing of ideas was enabled by a back-and-forth approach as they investigated the problem and explored possible solutions—an approach which continued throughout the research. A shared vision of a quality learning package to support student learning and safe practice was established on their willingness to participate in this cooperative process.

A critical dimension of reciprocity was the cooperative movement within and beyond the Project Team. The dimension and spirit of reciprocity was to underpin how this team, from their shared vision, interacted with the other groups within the project. Working across three sites was considered ‘next generation’ due to the extensive development of the Program beyond stakeholder consultation which required clear communication and commitment flowing from all involved.

Right from the word go … it wasn’t ‘we can’t do this’, it was ‘we need to do this, how can we do this’. (Nursing Director Interview 1)

A cohesive dialogue formed from the Project Team’s vision was joined by each group as they became accountable for their roles. While natural alignments existed within these groups, the Project Team facilitated a communication conduit so that timely iterative changes could be made.

This approach enabled groups and individual members to engage meaningfully with the work.

… so I think there was really good synergy between all three to try and get this product moving … so having a really engaged team from my perspective was a really strong enabler to get that done. (Nursing Director Interview 2)

Reciprocity kept the co-design and implementation process moving forward as there was a “genuine belief that working together gives insight” (Project Team Interview 3). It was a novel and innovative approach—the ‘next generation’ of building on network connections, ideas and learning together. This collaborative approach even in the design phase was shifting the conversation from previously siloed education packages across three institutions to partnering education and evolving the curricula so that nursing education could be relevant to industry, universities and RNs.

#### Flexible adaptations

The realisation of this deeper process raised awareness of boundaries and competing interests between university and industry. Expressing these boundaries required frank and honest conversations within the Project Team. These conversations occurred as team members recognised the complexity of the different structures within their institutions. Yet, while there was a readiness to address boundaries, for some members these disclaimers were “more revealing than anticipated” (Project Team Interview 1). The different interests and pressures from each institution meant that members were not free to make decisions early in the project.

… it is not that we don’t want to, but these are the boundaries within which we are able to work and recognising that we have different structures and processes for the different organisations that must be maintained. (Project Team Interview 1)

This brought an initial uncertainty as the team took time to reformulate their ideas, actively listen to each other, and then begin to recognise that they were using different terms to describe the same idea.

The Project Team continued this process of negotiating towards clarity with the other groups involved in the project. By giving opportunity for expression, members of the wider groups were able to explore the socio-political landscape and find a mutual space to collaborate rather than compete. This was vital as some protective elements were apparent in sharing knowledge and skills across the three institutions.

Everyone stepped back from their vested interest and looked at what could be the best outcome, so that we were getting a better product and better engagement. (Project Team Interview 3)

Significantly, as the work evolved, collaboration made use of each group’s strengths and resources. For industry, learning could occur close to care provision and intentionally focus on the student experience, while the access to a learning designer and lecturer from university created a professional learning package that utilised a multi-modal approach.

Flexible adaptations aligns with considering the right people for the right roles within the project. These deliberations required time, wisdom, and prudence as the Project Team wanted a whole and fulsome approach. Project Team members worked closely across all other teams, providing direction when needed and making space for other team members to flourish. This was specifically evident in the co-design of the education package in the Working Group. Within this group, education experts from each institution worked together to create an engaging quality postgraduate education product. While curriculum expertise was available from the Project Team, they did not impose on the Working Group. The same principles of trust, respect, communication, and connection to the vision harnessed the collective, expert knowledge of this group.

This nicely evolved. They worked exceptionally well to really focus on the student experience, to really focus on what was needed … everyone managed to step up because everyone could see the value in it and wanted to try things in a different way … I was really impressed with the standard. (Project Team Interview 4)

Flexible adaptations was also expressed as groups considered future directions, sustainability and replication following implementation. For industry, the Project Team recognised the need for greater enablement at the ward level to support sustainability. Some Industry Managers felt disconnected through limited communication, which affected their engagement. They were also concerned that the program impacted the integration of new staff to the ward.

My role was limited to releasing staff to attend their requirements. (Industry Manager Interview 2)

It was apparent that better lead times for clinical areas would support roster requirements and facilitate offline time for students, which hospital wards could manage. This would also enable webinar scheduling and assessment presentations to align with ward programs.

I think we need to think about different ways of being more flexible in how we do industry/academic engagement in ward time. (Project Team Interview 5)

At the university level, flexible enrolment options that match hospital intakes and nursing work also need to be considered. However, this would require a shift in the way university education is currently organised, since rigid enrolment processes do not currently meet the needs of students or industry.

#### Authentic learning (Education Program Partnership Group)

The Project Team intentionally sought to provide an environment for co-design where the blending of different expertise could transpire. The project’s inclusion of two hospitals was considered the ‘next generation’ of developing postgraduate education differently. This approach enabled working with practitioners and professionals who could bring authentic value to the design and implementation. The integration of their resources generated a substantive learning package from the new collegial relationships across the three institutions. What emerged was the development of a credible and highly valued product that met a “need in developing new to practice cancer nurses” (Nurse Educator Interview). Importantly, authentic learning facilitated the progression of sound theoretical grounding to the bedside.

It is nice when you can align your learning and theoretical components to the space where it happens in the workplace. (Nursing Director Interview 1)

While this work raised awareness of how industry can partner in the postgraduate education space, there were concerns regarding workloads for the Nurse Educators heavily involved in implementation and assessment. Conjoint positions, review and rationing of current work practices and sharing the financial burden could be future considerations. However, the industry partners understood the significance of their contribution to cancer nursing education in this co-operative approach.

I think it is organisations’ or industry’s responsibility and accountability to make sure that we set nurses up to achieve what they need to achieve with that academic flavour. (Nursing Director Interview 1)

Likewise, this approach meets the need of investing in safe patient care and nursing professionalism.

We need to be investing in our staff. We need to be investing in their education. We need to be investing in their career progression and further education and we all have an obligation in doing so. That is why I see this as being critical. The evidence tells us that a highly qualified educated workforce provides better outcomes. (Nursing Director Interview 2)

#### Authentic learning (program participants)

Program participants came to the course with an expectation of building their understanding and nursing knowledge of cancer care in order to practice safely. They anticipated a specific focus on grading, staging, pathophysiology and treatment to foster their workplace learning as a novice cancer nurse.

The modules are great for the novice nurse to gain a better understanding of patient and diagnosis. (Program Participant Interview 1)

The partnering approach provided a holistic learning package where program participants were supported to critique their knowledge, skills and practice and work towards assessment close to their daily work. It became a unique learning experience, with knowledge being consolidated through regular feedback sessions and onward opportunities for assistance and clarification. Access to specialist cancer nurses facilitated a sound understanding of the patient experience including diagnosis, testing and treatments.

… gave me a better understanding of the patient experience, how things work at the doctor’s end and allowed me to start thinking about how this might impact the patient at different stages, understanding better treatment options in relation to their specific disease and stage. (Program Participant Interview 4)

Interactive modules and assessment situated in the students’ context guided their education to generate experiences for further research. The case study presentations created spaces for the students to hear from each other’s experiences. Importantly, students linked their growing knowledge base to understanding the patient’s context. This supported practice confidence, interprofessional relationships and the ability to provide safe person-centred care.

…when my patient comes to have cancer treatment, I will look at the diagnosis and find out what stage they are. Then I will find out what type of diagnostic tests they needed to be diagnosed. Then link this to why the doctor has chosen the exact treatment for this specific patient. (Program Participant Interview 3)

For many involved, the partnership across the three institutions was considered a watershed. The university-industry-partnered education program accomplished an innovative approach to design and delivered an authentic postgraduate education program in cancer care services.

This partnership heralds the future of early nursing career development, linking industry and academia; and has the capacity to strengthen nursing professionalism. (Working Group action meeting note reflection)

Students also gained a relevant understanding of cancer nursing to support their care provision as novice practitioners. Notably, the sharing of new knowledge, resources and the new relationships established will be invaluable to future collaborations.

## Discussion

Developing nursing education programs in isolation prevents optimal creation of quality sustainable postgraduate education for the future nursing workforce. This co-creation requires a fundamental rethink of how industry and university partnerships work to deliver products that support student learning and enable safe, educated clinicians. Despite their mutual interest, there is a dearth of literature that explores how these two sectors can partner to deliver postgraduate education. Yet, recognising and working with the different perspectives from each group can facilitate a shared understanding and innovative solutions [18]. Co-creation requires each group to be comfortable to collectively engage with their divergent needs and understandings as they work towards a unified goal. As identified in this study, this understanding is dependent on the desire of each group to be in partnership and to see the benefit of the co-design initiatives: reciprocity. To realise the full potential of reciprocity, each party must intentionally relinquish control as they look for mutual transformative leadership within the partnership, which values doing postgraduate education more effectively [19]. Co-operation is enabled as the partnership recognises the need to investigate and act on their solutions and new shared interests through a genuine collaborative relationship [20]. Mutual ground can be achieved despite the challenges of considerable effort to form and sustain the partnership. The initial consensus building process can then be mentored to wider team members through the co-design work [21]. Importantly, ongoing delivery of agreed education can build on this foundation as the partnership is operationalised for future ventures.

The partners in this study recognised that existing postgraduate education processes are not beneficial or meeting nurses’ postgraduate education needs. As these partnerships take courage to appreciate and work with the nuances of each group, there is capacity to skilfully adapt to achieve an alignment of learning for all and enhance workplace safety. Building communication between the groups is essential for members to work with their strengths [22]. Our research established that excitement and growth were generated as members flattened the hierarchy, worked respectfully and recognised individual expertise. This benefit flowed beyond the initial team to the wider members, program participants and their organisations. There was also an *add-on* value of capacity-building which occurred as members worked closely across the two sectors. The Nurse Educators gained a deeper understanding of formal assessment requirements for the Australian Qualifications Framework as they worked with the university partner. University partners also raised collective awareness for what volumes of learning students and industry valued. Students created their own community of practice through the blended learning and close workplace learning approaches [23]. This was evident by student networking and engagement with online discussions, activities and feedback provided by the participant surveys. These unexpected outcomes warrant further consideration of the impact of the co-design approach.

The Framework enabled the partners to adapt to the complexity of the healthcare environment. Shared ownership within the partnership supported each Project Team member to use innovative ways of keeping their wider participants/groups connected and informed as the research progressed. As highlighted in the work of Gustafsson et al., [24], Project Team members’ movements among participants and partnership groups enabled a continual process of extending and retaining trust, which supported each group through unfamiliar processes. Project Team members’ strategies included clear communication, facilitating expertise, and working flexibly as proxies and stakeholders changed. In some cases, the Education Program Partnership Group participants expressed limited awareness of the postgraduate education program, yet Project Team members continued to creatively adapt, support and facilitate where necessary.

The University-Industry Integration Framework provides a unique guide for academic, industry and student connections in postgraduate education. While there are upfront development costs in co-designing a Program, it was designed to support industry to deliver education for beginning practitioners to be safe at the bedside. Once the Program is established, the ongoing revision and update is anticipated to occur only every three years. The ongoing profession and university benefit lies in the uptake of life-long learning and subsequent enrolment in postgraduation nursing courses. This research was supported with project funding, education providers may create opportunities through established industry relationships or new partnerships. A guide for University-Industry Integration (2021) was developed and is available from the Queensland Government website [25]. Future proofing these partnerships through a willingness to embed the framework may provide further direction. Building on existing programs across university and industry networks is an opportunity for further research.

### Implications

This research provides an exemplar for health services to initiate an effective partnership to improve education for novice nurses. High quality, relevant education in postgraduate nursing specialties cannot be effectively delivered by either universities or industry in isolation, this research provides implications for delivery of future sustainable education. The value of the framework guiding this project allow for adaptation to meet the needs of the partnership. That is, the industry partner can amend to join with any university provider, and the university may also deliver a co-design approach with private industry partners.

### Limitations of the study

The commitment and investment of established partnerships clearly enhanced this research, though this may not be the case for others embarking on similar ventures as trust and an understanding of ‘ways of working’ need to be established upfront. The timing of the program’s delivery was not flexible, and this created challenges for stakeholders. Short notice was impacted by advanced rostering of participants to clinical duties and limited preparation time and delivering the program early in the year meant that most of the work was undertaken over December and January. These are known to be busy times for health industries employing new graduates and for universities preparing for semester one courses in Australia.

Due to the small sample of program participants, results need to be viewed with caution and may not be generalisable for developing future postgraduate education programs. As purposeful sampling was used, we acknowledge this raises risk of bias and therefore reduces generalisability to all nurses. A further limitation is this research took place at two large metropolitan health services and a metropolitan university that were located in close proximity. This allowed some face-to-face work, which may have enhanced trust development. This research did not examine the costs associated with developing and delivering nursing education in partnership, and this is likely to be a determining factor in the replication and sustainability of this initiative. The study was undertaken through a global pandemic, so collaborations and deliverables were second to pandemic health service workloads and priorities.

## Conclusions

University and industry partners can apply the University-Industry Integration Framework [2] to achieve a shared vision, work in new ways, and deliver a successful postgraduate education program for cancer care services. Industry provides access and empowers staff to want to learn, while university academics provide teaching and learning integrity and credibility to education outputs. In a true co-design partnership as in this study, the reciprocal exchange of educational ideals was fostered for mutual benefit of university and industry [25].

Embarking on the postgraduate cancer care education program was not without the hindrances of workloads, timing, staff availability, completing legal contractual agreements in a timely way, and recruiting from unique health service areas. Further research needs to be undertaken to evaluate the benefits, financial and job satisfaction to stakeholders. Overall, stakeholders were highly satisfied with co-designing a postgraduate education program for cancer care nurses, leading to enhanced collaboration between university and industry partners and increased knowledge and skills in cancer care program participants.

### Electronic supplementary material

Below is the link to the electronic supplementary material.

Supplementary Material 1

## Data Availability

The datasets used and/or analysed during the current study are available from the corresponding author on reasonable request.

## Abbreviations

AQF: Australian Qualifications Framework
TEQSA: Tertiary Education Quality Standards Agency
