# Supplementary material for: Leveraging university-industry partnerships to optimise postgraduate nursing education
Source: BMC Nurs. 2023 Aug 4;22:256. doi: 10.1186/s12912-023-01419-1 (PMC10401860; doi:10.1186/s12912-023-01419-1)
Supplement: Supplementary file 1 — Supplementary Material 1 [file 12912_2023_1419_MOESM1_ESM.docx]

**
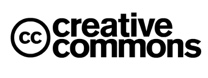
 (Additional file 1) UNIVERSITY-INDUSTRY INTEGRATION FRAMEWORK**

Theobald, K., McCarthy, A., Henderson, A., Coyer, F., Shaban, R., Fox, R., & Thomson, B. (2019). *Academic–industry integration in health: enhancing postgraduate professional learning [Final report 2019]*. Retrieved from Department of Education and Training website <https://ltr.edu.au/resources/SD15-5094_Theobald_FinalReport_2019.pdf>

CC BY-SA 4.0

**UNIVERSITY-INDUSTRY COLLABORATIVE PROCESSES**

1. Course sanctioned by ***senior leaders*** e.g. CEO, Executive Director, Head of School.
2. Broad agreement by ***middle management*** around optimum learning opportunities and relevant assessment, and where these types of opportunities are best accessed.
3. Operational level i.e. ***course co-ordinators and local leaders*** establish engagement plan, for example: capacity; level of engagement; resource implications. [i.e. staff in the workplace have a clear understanding of student learning outcomes and communicate this through shared collaborative conversations across university/ industry staff and students.]
4. Arrangements for building capacity across university and industry:

a. **Identify staff in the workplace** who can advance student learning. Clinicians, in the workplace, who take on *academic role e.g.,* ‘honorary lecturer’ must be qualified to at least one level of qualification higher than the course of study being taught, or have equivalent relevant academic or professional or practice-based experience and expertise to supervise and assess*.

b. **Plan the extent of the university ‘footprint’** in the clinical facility to significantly impact desired learning. This includes, numbers and types of positions e.g. ‘honorary lecturers, adjunct learning advisors’ (with agreed key performance indicators to ensure accountability to industry and university).

c. **Agree on supervision arrangements**, feedback processes, and assessment moderation/calibration activities. This needs to be collaboratively planned as it can directly affect staffing levels, rostering and skill mix in the clinical facility.

d. **Develop a communication plan** that outlines the nature, format, timing, and content of communication between university with industry that provides oversight and monitors these arrangements [e.g. weekly emails/ meetings to verify operations, scheduled feedback quarterly, reports of student progress (including impediments), evaluations of contribution of program]?

**UNIVERSITY**

Program/ course meets:

- Standards for higher education (Higher Education Standards Framework, 2015)
- Australian Qualifications Framework, 2013

**CURRICULUM**

**Unit 1&2 “*Advancing Clinical Practice I & II*”**

Higher order clinical care

**Unit 3 “*Transforming Clinical Practice*”**

Political, social, cultural, clinical trends

**Unit 4 “*Clinical Leadership*”**

Leading in health care

**INDUSTRY**

The *vision/values* statement of industry welcomes student participation and contribution;

*Contract/agreement* with University outlines specifics (refer to *communication plan* in processes);

Internal *policies* in place regarding workplace learning include placement, supervision and assessment regimens;

*Logistics implementation* plan e.g. identification of staff accountable for progress; time/opportunity for staff to supervise students, arrangements for enacting communication plan with university regarding student needs/progress and escalation of concerns;

*Recognition* program for staff who supervise.

**ADDITIONAL CONSIDERATIONS**

Establish criteria for involvement of persons (in university and industry) in decision-making and arrangements in service agreements. The responsible persons require authority, appropriate delegations, and time to be responsive to the agreed decisions. Time allocation to be reasonable to enact negotiated and agreed decisions.

**SUPPORTIVE MEASURES TO SUSTAIN PROCESSES**

1. *Industry staff* (i.e. honorary lecturer) receive adequate preparation and training commensurate to be able to facilitate expected academic and industry outcomes.
2. *The* *clinical practice team* (industry partners) is advised regarding students and assisted in the organisation of local work practices to advance student learning
3. *Staff who are proactive and successful* in advancing student learning are rewarded and recognised.
4. *Workload* is managed to facilitate staff’s ability to effectively interact with students.
5. *Communication process* between university and industry is timely, ongoing, and incorporates clarification of expectations of students’ ability and expected performance. It includes debriefing sessions, planning and review processes; feedback processes to others in the University and Industry (Henderson and Eaton 2013).

**UNIVERSITY**

Student completes course of study by meeting course expectations and the requisite standard of performance.

**STUDENT**

1. Student commitment and involvement in the development, delivery, and agreed outcomes of the course.
2. Open communication with university co-ordinator and clinical supervisors about student experience of learning to reflect, refine, and modify processes to best enable student to reach learning outcomes.

**INDUSTRY**

Safe practitioner that has the capacity to apply the outcomes of the course and is working toward increasing skilled clinical practice.

**STRUCTURES**

**PROCESSES**

**OUTCOMES**

**Feedback processes**
